# *PCD* Recognizes Outstanding Student Research: Patel et al on Emergency Medical Services Capacity for Prehospital Stroke Care in North Carolina

**DOI:** 10.5888/pcd10.130247

**Published:** 2013-09-05

**Authors:** Samuel F. Posner

This week, *Preventing Chronic Disease (PCD)* publishes the winning submission in the journal’s third annual Student Research Paper Contest, “Emergency Medical Services Capacity for Prehospital Stroke Care in North Carolina,” by Mehul D. Patel and colleagues (1). The contest is part of *PCD*’s effort to support and encourage the development of the next generation of public health professionals. Mr Patel is an epidemiology student in the Gillings School of Global Public Health at the University of North Carolina at Chapel Hill, and Dr Wayne Rosamond is his academic and dissertation advisor. As in previous years, submissions in the 2013 contest examined a range of topics and used a variety of analytic methods. Since the first contest in 2011, the quality of the submissions has improved, making the final decision that much more difficult for the panel of judges. 

As Patel and colleagues note, stroke morbidity and mortality is high in the United States and in North Carolina. It is well known that early treatment of stroke can make a difference in event outcomes. During the past decade, efforts have been made to raise public awareness of stroke signs and symptoms and the importance of acting early and to increase the knowledge and capacity of emergency medical services (EMS) to improve health outcomes. Patel and colleagues examined improvements in EMS capacity to identify and treat stroke in North Carolina from 2001 through 2012. A statewide survey in 2001 found deficiencies in EMS education and the use of protocols. The authors’ survey in 2012 documented major improvements since 2001, especially in prehospital stroke screening, and highlighted other areas that remain in need of improvement, including the use of destination plans.

This article nicely demonstrates the relationship among data, health, health care, and policy. The 2001 survey documented the need to improve the stroke care capacity of EMS systems in North Carolina, and surveillance data documented the burden of stroke (2,3). Together, this information supported the development and passage of state legislation in 2006 to address the availability of stroke-related resources among hospitals and EMS systems and the implementation of standardized EMS stroke care practices and policies. Results from the 2012 survey indicated that EMS had made progress in its capacity to provide services. The decline in stroke mortality in North Carolina documented by external data sources provides some ecological support to changes in health outcomes.

The study had one limitation that highlights an opportunity for collaboration between clinical and public health systems: the study had no direct measures of services provided, patient outcomes, or population-level indicators. This limitation points to a challenge faced by many public health researchers: access to health outcome data in addition to process and proxy outcome measures. Direct measurement of health outcomes is ultimately needed to determine intervention effectiveness and to make a convincing argument for public health programs and policies. In North Carolina, the decline in stroke mortality documented by external data sources parallels changes in policy and service protocols. However, Patel’s study did not include data on health outcomes or services provided, both of which could offer evidence that changes in health outcomes resulted from changes in policies and service protocols. Data from other sources can sometimes be used to triangulate findings; however, this approach has limitations. This gap in data highlights an opportunity for clinical and public health systems to work together to collect data that will provide the strongest evidence to evaluate policy changes.

Congratulations to Mr Patel and his colleagues on their outstanding work. We invite you to read the winning article (www.cdc.gov/pcd/issues/2013/13_0035.htm) and to listen to the accompanying podcast, an interview with the author. The study represents exceptional work, selected from a pool of several outstanding manuscripts, performed by members of the next generation of public health researchers. Although there can be only one contest winner, we were pleased to find that several manuscripts were of sufficient merit to warrant future publication in *PCD*. The review process for these student submissions was conducted by a panel of *PCD* editorial board members as well as Ellen Kersten, winner of the 2012 *PCD* Student Research Paper Contest. We look forward to the next round of student papers for the 2014 contest. The deadline for submission of student papers is January 31, 2014.
